# Establishing a pharmacotherapy induced ototoxicity programme within a service-learning approach

**DOI:** 10.4102/sajcd.v62i1.96

**Published:** 2015-06-17

**Authors:** Natalie Schellack, Anna M. Wium, Katerina Ehlert, Yolande van Aswegen, Andries Gous

**Affiliations:** 1 Discipline of Speech-Language Pathology and Audiology, Sefako Makgatho Health Sciences University, South Africa; 2 Department of Pharmacy, Sefako Makgatho Health Sciences University, South Africa

## Abstract

Pharmacotherapy-induced ototoxicity is growing, especially in developing countries such as South Africa. This highlights the importance of ototoxicity monitoring and management of hearing loss. This article focuses on the establishment of an ototoxicity clinic as a site for the implementation of a service-learning module in the Audiology programme. The clinic offers a unique opportunity of collaboration between pharmacists and an audiologist where pharmacotherapy-induced ototoxicity is uniquely monitored. The Sefako Makgatho Health Sciences University (SMU) provides training to both the disciplines audiology and pharmacy. The main aim of this article is to describe how ototoxicity monitoring is implemented in the curriculum within such an academic service-learning approach. Through service learning students develop a deeper understanding of course content, acquire new knowledge and engage in civic activity. It simultaneously provides a unique opportunity for interdisciplinary collaboration between the disciplines of audiology and pharmacy. The objectives for this programme are therefore to facilitate learning and to provide a service to the local community by identifying, preventing and monitoring medicine-induced hearing loss in in-hospital and out-patients; as well as to establish inter-disciplinary collaboration between the disciplines and stakeholders for more effective service delivery. The constant interdisciplinary teamwork between the audiologist, pharmacist, physician and nursing staff in the wards results in best practice and management of patients with ototoxic damage.

## Introduction

The creation of competent and caring professionals who contribute to the welfare of the country is the ultimate aim of higher education institutions (Stevens, 2009). Traditionally students were taught professionalism through their clinical training (Scudder, 2006). It is important that students not only obtain their clinical competence through the training of splinter skills that are based on theoretical knowledge, but that they learn by applying their knowledge, and to further become competent in analysing, synthesising, and evaluating what they have learnt in the classroom (Stevens, 2009).

The past decade has seen several changes in teaching and learning practices. One of the paths to facilitating professional competence of audiologists is though service-learning experiences. Academic service-learning (ASL) is a ‘valued pedagogy for engaged campuses’ (Bringle & Hatcher, 2009, p. 37) and is a methodology for teaching and learning that facilitates the application of knowledge and reflecting on it, while simultaneously addressing community needs. Through ASL, students develop a deeper understanding of course content, acquire new knowledge and engage in civic activity (Colby, Ehrlich, Beaumont & Stephens, 2003). This article focuses on a newly established ototoxicity clinic in a semi-rural context where students obtain hands-on learning experiences in ototoxicity monitoring by simultaneously providing a service to the community. The main aim of this article is to describe the collaboration between two disciplines (SMU – Department of Pharmacy and the Discipline of Speech-Language Pathology and Audiology) in the initiation of a pharmacotherapy induced ototoxicity clinic within a service-learning approach.

## Ototoxicity: The need for monitoring

South Africa is faced with a major challenge in terms of tuberculosis (TB), Human Immunodeficiency Virus (HIV) (Achkar, Kasprowicz & Wilson, 2011) and malaria (Department of Health, 2014). In all three of these conditions, hearing loss can be induced by the treating agents. Dr George Mukhari Academic Hospital (DGMAH) specifically encounters the challenges in treating TB and HIV. One of the challenges posed by these statistics lies in the fact that multi-drug resistant TB (MDR-TB) requires the use of an aminoglycoside for an extended period of time (Department of Health, 2014). Aminoglycoside-related ototoxicity has been well described in literature and is known to be irreversible (Cheng, Huth & Ricci, 2011). Therefore the TB burden in SA also accounts for an additional burden on the health care system of pharmacotherapy-induced ototoxicity and the resulting hearing loss in patients with TB.

Specific attention should further be paid to patients who are treated with ototoxic medications, as they are at risk for hearing loss. Patients suffering from multi-drug resistant tuberculosis, renal failure (Schacht, Talaska & Xie, 2011), oncology patients (Handelsman, 2011), and neonates with severe infections or sepsis who are treated in the neonatal intensive care unit (Cheng *et al*., 2011) can be classified as high-risk patient populations for possible ototoxicity.

According to Bratt *et al*., (2005) the best way to detect ototoxicity is by assessing auditory and vestibular function directly. Early detection of ototoxic damage can improve the treatment outcome through minimising hearing loss progression and by counselling and rehabilitation of the patient. It also provides the physicians the opportunity to adjust the therapeutic regimen to the benefit of the patient. An effective ototoxicity monitoring programme should consist of various aspects, including specific criteria to identify ototoxicity, timely identification of the at-risk population, pre-treatment counselling regarding the potential ototoxic effect of the drug treatment, valid baseline measurements and on-going monitoring during and after the completion of the drug treatment (ASHA, 1994).

## What is ototoxicity?

Ototoxicity can broadly be defined as the tendency of certain substances, either systemic or topical, to cause functional impairment and cellular damage to the tissues of the inner ear and especially to the end organs of the cochlear and vestibular divisions of the eight cranial nerves (Roland & Rutka, 2004).

Medications that may lead to ototoxicity can either cause cochleotoxicity or vestibulotoxicity, depending on their mechanism of action in terms of the ototoxicity. Certain medications, e.g. aminoglycosides, have the tendency to cause both (Selimoglu, 2007). Cochleotoxicity may present as reversible or irreversible hearing loss, tinnitus, hyperacusis, as well as the difficulty in system may present as disequilibrium, unsteadiness when walking or ataxia, oscillopsia, nystagmus and/or vertigo (Selimoglu, 2007).

Blockman and Sinxadi (2009) consider a drug to be ototoxic if it has any toxic effects on the structures of the inner ear, such as the cochlea, vestibule, semi-circular canals and the otoliths. Hearing problems can start within minutes to several days after the administration of an ototoxic drug, or it is possible that it can only start presenting several years after treatment with ototoxic medicine (De Andrade, Hajat & Khoza-Shangase, 2009). Depending on the type of medicine being used as part of the patient’s specific treatment regimen, hearing loss may be reversible or permanent (irreversible). Examples of drugs causing irreversible hearing loss are aminoglycosides, platinum compounds and quinine. Reversible hearing loss can occur as a result of loop diuretics, salicylates and non-steroidal anti-inflammatories (Blockman & Sinxadi, 2009).

Considering that most primary care clinicians and clinical pharmacists have seen patients who have suffered from ototoxicity at some point in their career, it makes sense that audiologists should be more involved in a collaborative patient education effort with physicians, nurses and pharmacists concerning ototoxic medications (Banotai, 2004). Pharmacists can play an active role in identifying and monitoring ototoxicity agents, and can subsequently, where applicable, perform therapeutic drug monitoring to reduce ototoxicity (Schellack & Naude, 2013). Audiologists often only become involved in ototoxicity-related problems after the patients stop taking medication or leave the hospital and experience hearing or vestibular problems.

## Establishing an ototoxicity clinic as service-learning context

The establishment of the pharmacotherapy-induced ototoxicity programme ensures that there is a team approach from the clinical pharmacist, audiologist, nurse and physician in managing the patient. This service to the local community (i.e. the hospital) created a platform for an academic service-learning approach (ASL), which is a form of experiential learning.

A service-learning approach places equal emphasis on student learning and service to the community (Bringle & Hatcher, 2009). Simultaneously, it aims to facilitate social responsibility. It is a process through which students develop knowledge, skills and values from direct experiences outside a traditional academic setting. Well-planned supervised and assessed service-learning programmes promotes interdisciplinary learning, facilitates social responsibility, career development, cultural awareness, leadership and other professional and intellectual skills.

SMU provides training to both the disciplines audiology and pharmacy, and is attached to the Dr George Mukhari Academic Hospital (DGMAH), which is adjacent to the campus. Previously, there were no accepted clinical protocols or criteria for ototoxic change using objective measures of ototoxicity at DGMAH. To this effect, two academic departments at SMU (Department of Pharmacy and the Discipline of Speech-Language Pathology and Audiology) and one clinical department (Speech-Language and Audiology Department of Dr George Mukhari Academic Hospital) initiated an ototoxicity clinic. The establishment of a programme with medicinal and audiological monitoring for ototoxicity provided the basis for developing objective protocols for monitoring ototoxic damage. The aim of the collaboration was to engage in service-learning at the Dr George Mukhari Academic Hospital by establishing a clinic to monitor pharmacotherapy-induced ototoxicity. Through such a clinic a service could be provided to the patients at DGMAH by identifying, preventing and monitoring medicine-induced hearing loss to in-hospital and out-patients. The objectives for this project were aligned with the criteria for ASL (Stacey, Rice & Langer, 2001) as follows:

- The *service provided is relevant and meaningful* to the hospital community, the students and the academic staff. Ototoxic monitoring services have not been available to this hospital prior to the establishment of the ototoxicity clinic, and have now become an integral and necessary part of more effective service delivery. During regular staff-student meetings in the clinic, the nurses and doctors in the various units value the contribution of the students and consider their role as necessary. Effective service delivery requires effective inter-disciplinary collaboration between the disciplines and stakeholders in order to provide more effective service delivery in health care. Literature states that audiologists should collaborate with doctors, nursing staff and other health care professionals when monitoring a patient presenting with ototoxicity, but in current practises and existing programmes audiologists work on their own and only provide results and reports to other professionals in an inter-disciplinary approach. (Dille *et al*., 2011).
- The experience *enhances academic learning* as it strengthens the accomplishment of module outcomes. The objectives related to patients are considered service-learning (S-L) outcomes in the curriculum. This experience facilitates interdisciplinary learning between students in the disciplines audiology and pharmacology, to obtain competence (knowledge and skills) in managing pharmacotherapy induced ototoxicity. This allows audiology students the opportunity to apply their knowledge regarding ototoxicity monitoring and to reflect on it (Bender, Daniels, Lazarus, Naude & Sattar, 2006).
- The rotation in the ototoxicity clinic fosters students’ *social responsibility* as the knowledge, skills and values obtained prepare students for active involvement in future communities (Bender *et al*., 2006)
- The students engage in structured *reflection* by completing their reflection journals to think critically about what happened at the service site and their experiences at the end of each day. They need to do this in order to plan for the next session. Through this reflection they examine their own feelings, attitudes and beliefs. They further relate their learning to the module outcomes and apply their prior knowledge to conduct a more effective service in future. At the conclusion of the rotation block they complete a course evaluation sheet to evaluate the entire experience.

Considering the *Health Professions Act 56 of 1974* for clinical training of students in audiology, it was necessary to include aspects of prevention and identification; assessment; and management of ototoxicity. This early detection of ototoxicity will make it possible to prevent the progression of hearing loss to lower frequencies where speech perception can be greatly impaired. The Department of Pharmacy provides a service of evaluation and possible identification of medicines that has the potential of causing ototoxicity; with subsequent monitoring of the medicine in terms of dose, duration, levels (peak and trough) where indicated, and renal function. Where appropriate, dosage adjustments are made and medicines are stopped or changed for less toxic agents. The department also developed a short course that is available on line to promote education in the monitoring of ototoxicity. Pharmacy postgraduate students are also enrolled to investigate pharmacotherapy-induced ototoxicity. To this effect they collaborate with the discipline of speech-language pathology and audiology for audiological monitoring with possible identification of hearing loss. Educational activities need to be evaluated to ensure that trainees will professionally benefit from them. Monitoring is achieved through interdisciplinary team meetings and patient feedback. Reflective journals kept by students facilitate higher level learning as they are expected to analyse and synthesise the information learnt within ototoxicity as well as prior knowledge needed from other audiological modules. The criterion for judging programmes is the extent to which it can be used to make decisions that improve the programme, which implies that the findings are valued and credible.

Students engage in the service-learning activity in a rotation format and the process consists of several phases: preparation, acting (service), reflecting, and lastly evaluating whether the learning outcomes and service goals were met (Bender *et al*., 2006). The latter contributes to the effectiveness of the service provided. Students are initially orientated for their ASL experience firstly in the classroom by the pharmacists on the topics of pharmacotherapy and ototoxicity. Secondly they are prepared by their supervisors at the clinic to become familiarised with learning outcomes and the assessment procedures.

Several aspects had to be considered in the establishment of such a clinic as a service-learning context (Ginsberg, 2007): Firstly, the learning goals in the curriculum from which students could benefit from hands-on learning experience had to be considered. These included those goals where direct interaction with clients was concerned. Secondly, the specific context where these goals could be realised had to be identified. In this case, an academic hospital in a rural context that was within easy access for students appeared to be the most suitable choice. As no ototoxicity monitoring services have previously been available in this context, the patients in specific wards (NICU, paediatrics, renal, TB and oncology) could certainly benefit from such an incentive. In establishing this clinic as a vehicle for service-learning, it was further necessary to consider the types of activities that the students had to perform. With regards to ototoxicity, these include identification of the medicines prescribed and the basic mechanisms through which certain medicines can cause ototoxicity, case history, employing an audiology protocol to assess hearing abilities with regards to baseline testing and serial monitoring, designing and implementing a management plan, report writing, ethical decision-making, counselling appropriate to a variety of audiences with different levels of knowledge and collaboration with the pharmacist, physician and nurse. Knowledge from other audiology modules within the Audiology curriculum is needed to achieve the outcomes in ototoxicity monitoring. The critical cross-field outcomes within the curriculum need to be applied and integrated. These include:

- Identifying and solving problems through responsible decision-making and creative, critical thinking.
- Working effectively with others as a member of a team, group, organisation and community.
- Organising and managing oneself and one’s activities responsibly and effectively
- Collecting, analysing, organising and critically evaluating information.
- Communicating effectively using visual, mathematical and/or language skills in the modes of oral and/or written persuasion.
- Demonstrating and understanding of the world as a set of related systems by recognising that problem-solving contexts do not exist in isolation (inter-disciplinary collaboration).
- Using science and technology effectively and critically, showing responsibility toward the environment and health of others.
- Developing entrepreneurial opportunities.
- Contributing to the full personal development of each learner and the social and economic development of the society at large, by making it the underlying intention of any programme of learning to make an individual aware of the importance of:
  - reflecting on and exploring a variety of strategies to learn more effectively
  - participating as responsible citizens in the life of local, national and global communities
  - being culturally and aesthetically sensitive across a range of social contexts.
  - looking into a variety of educational and professional opportunities.

These activities could not only provide a service to the wards in the hospital, the patients and their significant others, but also provide students with an opportunity to learn (Altosino & Armstrong, 2014).

The process of establishing the ototoxicity clinic required the following:

- *Human resources:* Staff from both the Pharmacy and Speech-Language Pathology and Audiology departments are involved in the clinical training. One supervisor from the Discipline of Speech-Language Pathology and Audiology manages the clinic on a weekly basis. Whenever the students are not available (e.g. during examination periods and recess), this role is taken over by the staff from the DGMAH Speech Therapy and Audiology Department.
- *Site and physical space*: The clinic is situated in the Skills-lab at Medunsa Campus and testing is conducted in the paediatric oncology and renal wards at DGMAH. The clinic is situated in an ideal quiet environment for audiology testing procedures. Challenges are experienced when hearing screening takes place in the wards due to noise levels. Air-conditioning is available in the clinic, which improves infection control, as infections spread quicker in a heated environment (HICPAC, 2003).
- *Technology and protocol:* Baseline and serial monitoring of pure-tone hearing thresholds (with a frequency range of 250 to 8000 Hz) using serial audiograms are the most effective indicators of hearing loss, especially when ultra-high frequency testing is included (Bratt *et al.*, 2005). Extended high-frequency (EHF) and otoacoustic emissions (OAEs) are audiologic tests that are sensitive to primary ototoxic damage. EHF and OAEs can detect any changes in the auditory function before ototoxicity affects the hearing at such frequencies where speech recognition can be affected (Kraemer, Knight, Neuwelt & Winter, 2007). OAEs evaluate the cochlear outer hair cell system. Early changes in OAE may reflect on cochlear damage that could progress to further hearing loss if the ototoxic medication is continued. The *audiological services* available at the clinic provide baseline and serial monitoring of pure-tone thresholds (with a frequency range of 250 to 8000 Hz) in a soundproof booth, tympanometry, as well as distortion product (DPOAE) and transient evoked oto-acoustic emissions (TEOAE). Unfortunately, EHF audiometry is not available due to a lack of appropriate equipment in the clinic which is due to a lack of funding. When testing is done in the wards at DGMAH, only OAE testing and tympanometry is done, as equipment used for baseline and serial monitoring of pure-tone hearing thresholds is not mobile.
  - Often, hospital treatment protocols have specific time points for performing audiological assessments. These times points are based on several factors, such as diagnosis, treatment plan, patient age and ototoxicity risk (Bass, White & Jones, 2013). At the SMU clinic the patients are evaluated at any time during or after therapy if a noticeable change in hearing occurs. Bass *et al*. (2013) is of the opinion that patients diagnosed with severe or fluctuating hearing loss should be followed up more frequently than the ototoxicity protocol indicates, at least until hearing loss stabilises. In the Medunsa clinic ototoxicity monitoring is not concluded at the end of therapy, as delayed onset and progressive hearing loss has been documented in patients treated with ototoxic medication (Bass *et al*., 2013).
  - To date no accepted clinical techniques for vestibulotoxicity exist despite reported cases of possible effects thereof (Jones, 2008). Patients that display symptoms such as dizziness and unsteady gate associated with ototoxic therapy are referred for a complete vestibular evaluation at SMU Vestibular Clinic.
- *Needs of stakeholders:* Funding is a major need for the clinic, as no funds were available at the onset of the programme. Up to the present, existing equipment from the Discipline of Speech-Language Pathology and Audiology had to be utilised. This aspect poses occasional challenges, as the equipment is also needed at several other student training sites. Although EHF audiometry is a vital test to be administered in an ototoxicity monitoring programme (Kraemer *et al*., 2007), this equipment is not available at present.

## Challenges and strengths

OAEs are successfully used for evaluating cochlear outer hair cell function (Kraemer *et al*., 2007). Many adults with normal hearing present with low-level OAEs. Therefore, in ototoxicity, the ability to detect low-level OAEs and small OAE changes over time is required. Achieving valid and reliable measurements of low-level OAEs pushes the limits of standard OAE equipment, which invariably has been designed with infant screening in mind, as infants with normal hearing usually present with large OAEs and are easy to elicit. Eliciting low-level OAEs are also more affected by environmental noise or noise generated from a restless patient. Despite these challenges, OAEs can be successfully used in ototoxicity programmes (Marshall & Miller, 2007).

Another challenge is that some patients (e.g. the paediatric oncology patients) are too ill and immunocompromised to attend the clinic. This results in only OAEs being conducted, as baseline and serial measures are not possible in the wards due to immobile equipment and noise levels. Therefore, pure-tone thresholds, which is considered as the most effective measure of hearing loss is not possible. OAEs are conducted at the patient’s bedside or in a section of the ward. The acoustic climate of the wards is noisy, which makes is difficult to obtain reliable results on a repeated basis. Nursing staff also do not always realise the importance of a quiet environment during OAE testing.

The collaborations allows for the following strengths and weaknesses:

In terms of the former, a definite strength of this ototoxicity programme is the constant interdisciplinary teamwork between the audiologist, pharmacist, physician and nursing staff in the wards. Effective teamwork based on evidence based practices may enhance better practices and management of patients with ototoxic and vestibulotoxic damage.

In addition, there is a close proximity by providing easy and frequent access to the ototoxicity clinic; there is a clear communication and trust between the two disciplines; the patient sees both disciplines at once, which proved to be more effective than seeing the patient one after the other, and this increases the communication between the disciplines and the patient. The patient receives more effective treatment because the concerns are addressed immediately and ototoxic effects can be minimised and/or prevented if possible. State of the art equipment that is frequently calibrated is available to increase reliability. Student training is also taking place within a service-learning context. The hospital staff is also involved, which ensures the continuation of the ototoxicity clinic services when the university is closed for recess.

In terms of weaknesses, the student schedule causes the clinic to run according to the university calendar, which causes an increased workload for the hospital staff. Testing often occurs in the wards due to the patients being immunocompromised. The noise levels in the wards are high, which makes the testing challenging and often creates unreliable results. There is a lack of equipment for high-frequency audiometry at present, which ideally should be part of the protocol.

### 

#### Experiences of the stakeholders

At present there is no formal evidence of the experiences of the stakeholders, as this matter currently is being researched. The staff from the hospital departments expressed appreciation for the service and continues to send patients for screening. The clinical pharmacists have established working relationships with the physicians and audiologists and have become familiar with the type of hearing impairment caused by the different drugs. The audiologists performing the monitoring find the wards to be too noisy at times and struggle to obtain reliable OAE results. Although a quiet environment is the ideal for efficient testing, this is not necessarily the case. In reality the noise levels are high, which often cause the results to be unreliable and patients have to be re-assessed. In addition, the procedure takes much longer than it usually should. Patients who are not too ill are transported to the ototoxicity clinic where the environment is more conducive to testing. Student training has been successful, as students perform well in conducting the assessment procedures. Students attend on a rotation basis and block review reports and reflective journals have shown a positive result in terms of student self-review of competencies.

#### Content taught

The ototoxicity clinic at SMU provides a platform for student teaching and learning within a service-learning approach. Audiology students have the opportunity to work within an interdisciplinary team with pharmacists, nurses and physicians, and to interact with a diverse group of patients. The Department of Pharmacy provides the students with the background information on pharmacology and drugs that cause ototoxicity in the classroom prior to the onset of their rotation. On clinic days the students provide ototoxicity-monitoring services, including counselling to adult and paediatric patients affected by ototoxic and vestibulotoxic medications. Students are also required to develop a management plan for patients identified with ototoxic and vestibulotoxic damage. These plans are also submitted for assessment. Report writing is a collaborative effort between the clinical pharmacist and the audiologist. Reports are based on a discussion of results and planning of the way forward for each patient. While providing a vital service to the community, students are able to meet their learning outcomes related to ototoxicity monitoring within a hospital setting.

Summative and formative assessment procedures are applied throughout the clinical block. At the end of the module, the students are able to conduct an age and case-appropriate interview through the selection of appropriate questions from a case history form, display well-developed information retrieval skills together with the ability to critically analyse and synthesise information obtained from the assessment. Students are also expected to conduct and apply age- and case-appropriate protocols during the conduction of the basic audiometric test battery, critically analyse and synthesise information and interpret accurately. Additionally, students are able to conduct and apply age and case-appropriate procedures during the determination of diagnostic OAEs displaying logical and critical thinking; as well as demonstrate engaged participation during discussion of results. Students are expected to summarise audiometric results and explain it in a well-structured comprehensible manner during feedback and in the diagnostic report to the client, the parents, caregivers and/or significant others. Reflective journals allow analysis and synthesis of information learnt. When working with nurses, doctors, pharmacists and patients in the clinic, the students are exposed to multidisciplinary teamwork.

## Role of the facilitators

One staff member from the Pharmacy and Audiology Department, assisted by a Masters student, provides consultations with regard to the ototoxic medication. They also brief the students in Speech-Language Pathology and Audiology regarding ototoxicity, pharmacotherapy and the clinic protocol. Demonstrations in the use of the equipment for students training in audiology are offered and supervision is provided, whilst students gain hands-on testing experience. The entire process of learning and serving is monitored through structured assessment instruments by the facilitators.

## Attendance, and learning and teaching opportunities

From the initiation of the clinic in February 2013 up until April 2014, a total of 52 patients with 102 patient visits (an average of 1.9 visits per patient) attended the clinic. Some patients visited the clinic more than once depending on the recommendations by the team. All of these patients had a regimen containing either one or two medicines that may cause ototoxicity. To date 48 students have been trained in the clinic, and from their course evaluations and reflections on the module it appears as if they consider their training as good or excellent.

## Logistics

The clinic is conducted on Friday mornings between 08h00 and 12h00. Currently patients are referred by the paediatric oncology unit, NICU, renal and internal medicine wards. The clinical pharmacist identifies patients from other unit’s e.g. internal medicine. The programme intends to extend to other departments, e.g. adult oncology. Patients with pharmacotherapy that may cause ototoxicity are either identified by the clinical pharmacist or referred to the service department (which is the hospital department of Pharmacy or Speech-Language and Audiology). The clinical pharmacist then provides a description of the type of toxicity to be expected (i.e. vestibular/cochleotoxic and reversible/irreversible toxicity). The audiologist then assesses the patient and a full report is written from both disciplines with recommendations for alteration of therapy to the treating physician. The current regimen of prescription contains either one or two medicines that may cause ototoxicity. The medicines mostly implicated include amikacin, gentamicin, cisplatin and furosemide (at high dosages and used orally for extended periods of time in patients within the renal unit). These medicines might have been used alone or in combination. Additional details on the type of ototoxicity seen with these agents will be included as part of a Master’s degree study currently being conducted at the clinic.

## Conclusion

Through a service-learning approach, it is possible to form partnerships with the hospital staff and other stakeholders in order to meet the hospital community’s needs, but also to meet the specific learning outcomes, of which inter-professional collaboration and teamwork is but one. Through ASL a reciprocal relationship is established in which both parties benefit.

The establishment of this service-learning programme has created a unique opportunity for interdisciplinary collaboration between audiologists and pharmacists. Such collaboration provides an opportunity for early identification of medicines that may cause hearing impairment in vulnerable patients. Early identification affords early intervention for both the clinical pharmacist and the audiologist. Future research should further explore the nature and extent of hearing loss caused by the various agents and the experiences of stakeholders. A service-learning approach seems to contribute to the quality of their training.
